# Statistical Assumptions in Orthopaedic Literature: Are Study Findings at Risk?

**DOI:** 10.7759/cureus.18694

**Published:** 2021-10-12

**Authors:** Anthony V Christiano, Daniel A London, Joseph P Barbera, Gregory M Frechette, Stephen R Selverian, Amy S Nowacki

**Affiliations:** 1 Department of Orthopaedic Surgery, Mount Sinai Health System, New York, USA; 2 Department of Quantitative Health Sciences, Cleveland Clinic, Cleveland, USA

**Keywords:** statistical assumptions, orthopaedic surgery, regression analysis, statistical analysis, study validity

## Abstract

Background

As orthopaedic surgery becomes more evidence-based, the need for rigorous research has increased. This results in more complex studies that employ more sophisticated statistical analysis, often some form of regression. These statistical techniques require the data to meet certain assumptions for the findings to be considered valid. The purpose of this study is to determine the common regression techniques employed in the orthopaedic surgery literature, and demonstrate how often the assumptions of regression analyses are met and reported.

Methods

Studies published in the Journal of Bone & Joint Surgery (JBJS) in 2017 and 2018 were reviewed. Commentaries, editorials, and systematic reviews were excluded. The statistical analyses performed in each study were documented. When regression analyses were utilized, the article was reviewed for evidence that the necessary assumptions underlying the statistical methodology were assessed and met.

Results

From the 470 studies that were reviewed, the most common statistical test reported was the independent-samples t-test (n=215, 45.7%). Also, 201 studies (42.8%) implemented some form of regression analysis. The most common regression was a logistic regression (n= 106). None of the 201 studies using regression analysis reported meeting all of the necessary assumptions to appropriately use a regression test.

Conclusion

Many recent studies published in JBJS depended on regression analyses to reach their conclusions, but none fully reported the necessary assumptions of these tests. Orthopaedic surgery journals should be more transparent in reporting the methodology of statistical tests, and readers must beware of possible gaps in statistical methodology and critically evaluate the studies' findings.

## Introduction

To facilitate the analysis of larger clinical data repositories and to derive meaningful conclusions, there is increasing use of complex inferential statistical methods [1,2]. Modeling the relationship of one variable to another is often estimated using regression analysis to adjust for the effect of other variables on the response of interest. With an assortment of options, choosing an appropriate statistical methodology is based on the research question being asked and the types of data involved (continuous, binary, count, etc.). In order for any statistical test to be properly implemented with reliable findings, certain data assumptions must be met, and these underlying assumptions differ for every statistical test [3]. Failure to meet these assumptions can result in inaccurate results, which is problematic for many reasons. In particular, when conducting hypothesis testing it can result in both false negatives and false positives, depending on the particular assumption being violated. However, these assumptions may not always be reported in the orthopaedic literature, thereby making it challenging for readers to properly critique the validity of a manuscript’s stated results.

Furthermore, there is a precedent for concern of statistical validity in the orthopaedic literature. For example, Lebrun et al. in their review of 866 studies in American Journal of Sports Medicine demonstrated that while 135 studies analyzed dependent observations, 111 studies (82%) failed to account for non-independence, calling into question the validity of clinical claims based on that data analysis [4]. Furthermore, orthopaedic studies have been shown to be quite fragile - meaning that changing the outcomes of only a few patients from a non-event to an event could change the statistical significance [5]. Such findings increase the importance of having researchers performing correct statistical tests based on the nature of their data, and demonstrating that appropriate statistical assumptions are met.

The purpose of this study is to review recent studies published in the Journal of Bone and Joint Surgery (JBJS), to categorize the statistical analyses utilized in each study, and to then determine if the necessary statistical assumptions associated with regression techniques are reported. JBJS was selected due to its elevated status in the orthopaedic literature, often considered a gold standard, as well as its employment of separate methodological/statistical reviewers as part of its standard peer-review process. We hypothesized that less than 50% of the orthopaedic literature that employs regression analyses would fully report on the necessary underlying assumptions that must be met to appropriately use regression methodology.

## Materials and methods

IRB approval was not needed in order to conduct this retrospective study, as all data was obtained from existing articles that are publicly available. All studies published in JBJS in 2017 and 2018 were reviewed. Commentaries, editorials, meta-analyses, and systematic reviews were excluded. The statistical analyses performed in each study were documented and divided into unadjusted and regression-based methods (Table 1). When regression techniques were used, the article was reviewed for objective evidence that the necessary assumptions underlying the statistical approach were assessed and met (Table 2), including either a direct statement that a test was conducted or the actual results of the necessary test. This list of necessary assumptions was derived from a consensus amongst the authors with advanced training in statistical methodology (DL and AN). Each of these authors derived their own list of necessary assumptions independently; the two lists were then compared. All common assumptions were included. Additional methodologies exist for checking various assumptions and all were counted as evidence of assumption checking, however, only the most common are included in the tables. Methods based on univariable or bivariable tests of association (unadjusted) are classified as unadjusted. Methods with the capability to adjust for covariates are classified as regression. Underlying assumptions need to be met for both unadjusted and regression-based methods of analysis. However, the authors decided to not assess the assumptions underlying unadjusted methods. This decision was based on the fact that these methodologies (and thus assumptions) are well known to researchers and readers and relatively elementary. It is of the authors’ opinion that it is not unexpected for these assumptions to not be explicitly stated within a manuscript. Instead, the authors’ focus was on more advanced methods that rely on a higher degree of underlying statistical assumptions and may not be as familiar to the average reader or researcher.

**Table 1:** Listing of statistical analysis methods based on their categorization of being unadjusted or regression based methodologies and their frequency in the orthopaedic surgery literature.

Unadjusted Methods	No. of Studies			Regression Methods	No. of Studies	
Independent samples t-test	215	45.7%		Linear regression	79	16.8%
Mann-Whitney/Wilcoxon rank sum test	103	21.9%		Logistic regression	106	22.6%
Chi-square test	136	28.9%		Poisson/Negative binomial regression	4	0.9%
Wilcoxon signed-rank test	32	6.8%		Mixed model regression	0	0.0%
Fisher’s exact test	104	22.1%		Cox proportional hazards regression	38	8.1%
One-way ANOVA test	93	19.8%				
Kruskal-Wallis test	37	7.9%				
Kaplan Meier/Log-rank test	60	12.8%				

**Table 2:** Listing of statistical assumptions that were assessed according to type of regression model.

Statistical assumptions assessed according to the type of regression model
General for all regression models
No multicollinearity: correlation coefficients
No multicollinearity: variance inflation factors (VIFs)
No multicollinearity: tolerances or condition indices
Linear regression
Continuous outcome
Independence of observations via study design
Independence of observations via a residual time series plot
Independence of observations via Durbin-Watson test
Independence of observations via plot of residual autocorrelation
Residuals normally distributed via a histogram
Residuals normally distributed via a Q-Q plot
Residuals normally distributed with a goodness of fit test (Kolmogorov-Smirnov test, Shapiro-Wilk test)
Constant variance: plot of residuals vs. predicted values
Constant variance: plot of residuals vs. independent variables
Constant variance: Goldfeld-Quandt test
Linear relationships for numeric variables: scatter plots
Linear relationships for numeric variables: plot of observed vs. predicted values
Linear relationships for numeric variables: plot of residuals vs. predicted values
Linear relationships for numeric variables: plot of residuals vs. individual numeric predictor
Avoid overfitting of model: 20 subjects per regression coefficient
Logistic regression
Binary outcome
Independence of observations: study design
Linear in the logit for numeric variables: LOESS smoother
Linear in the logit for numeric variables: scatter plot between numeric predictor and the logit
Investigate model fit (Hosmer-Lemeshow goodness-of-fit test, Pearson/Deviance residuals)
Avoid overfitting of model: 10 events (non-events) per regression coefficient
Poisson/Negative binomial regression
Count/rate outcome
Independence of observations: Study design
Poisson → mean = variance: mention of overdispersion
Poisson → mean = variance: Pearson dispersion statistic
Poisson → mean = variance: compare residual deviance with degrees of freedom
Consideration of an offset term
Avoid overfitting of model: 20 subjects per regression coefficient
Cox proportional hazard regression
Time-to-event outcome
Non-informative censoring: study design
Independence of observations: study design
Linear in the log-hazard: plot partial sums of martingale residuals
Proportional hazards: examine log(-log(S(t))) plots
Proportional hazards: interaction with time in the model
Proportional hazards: score residuals
Proportional hazards: Schoenfeld residuals
Avoid overfitting of model: 10 events per regression coefficient

The authors manually reviewed each article. Articles were divided equally amongst the authors and in a random fashion. Reviewers paid particular attention to the methods and results sections and if any item from Table 1 or Table 2 were mentioned throughout the manuscript, then that paper was considered to have met that specific criterion.

Descriptive analyses of our data were performed to determine the frequency of use of each type of statistical methodology, as well as the frequency in which underlying assumptions were reported. Finally, each manuscript’s analyses were categorized as either acceptable or potentially over-fit, depending on where or not the models used contained less than the recommended number of subjects/events per regression coefficient. To perform these descriptive analyses, no underlying statistical assumptions are required.

## Results

For this study, 470 studies met our inclusion criteria and were reviewed, and 408 studies employed unadjusted or regression analyses in their manuscript (86.8%). The overall most common statistical test performed was the independent samples t-test (n=215, 45.7%). Utilization of the unadjusted tests can be seen in Table 1. Furthermore, 201 studies (42.8%) used a regression methodology. The most common regression method employed was a logistic regression (n= 106, 22.6%). Utilization of the various regression methods can be seen in Table 1. None of the 201 studies using any type of regression analysis reported meeting all of the necessary assumptions to appropriately use a regression technique. Additionally, a limited range of 0% to 25% of studies report meeting at least one underlying assumption for the regression technique utilized. Moreover, examining the general assumption of no multicollinearity that underlies all model types, only 13 studies (6.5%) reported on checking for this potential issue. Also concerning is that over 35% of all regression models did not have enough participants or events for all the regression coefficients included, which raises concern for over-fitting of data. The frequency of assumptions being met and reported, as well as concern for over-fitting of data can be seen in Table 3.

**Table 3:** Frequency of manuscripts meeting all assumptions, at least 1 assumption, and concerns for over-fitting of data according to type of regression model.

Regression Methods	No. of Studies	Meeting ALL assumptions	Meeting at least 1 assumption	Concern for overfitting data
Linear regression	79	0	20 (25.3%)	29 (36.7%)
Logistic regression	106	0	13 (12.3%)	42 (39.6%)
Poisson/Negative binomial regression	4	0	0 (0%)	0 (0.0%)
Mixed model regression	0	---	---	---
Cox proportional hazards regression	38	0	7 (18.4%)	14 (36.8%)

The strength of multivariable regression analysis is its ability to determine how multiple independent variables, which are related to one another, are related to an outcome. Multicollinearity occurs when these independent variables are highly correlated to one another. The interpretation of a regression coefficient is that it represents the mean change in the outcome for each one-unit change in an independent variable when you hold all of the other independent variables constant. The theory is that you can change the value of one independent variable and not the others. However, when independent variables are correlated, it specifies that changes in one variable are associated with shifts in another variable(s). It becomes difficult for the model to estimate the relationship between each independent variable and the outcome independently because the independent variables tend to change in unison. In the presence of multicollinearity, problems arise such as biased coefficient estimates, inflated standard errors, and thus a loss of power [6]. Multicollinearity affects the coefficients and p-values, but it does not influence the predictions, precision of the predictions, and the goodness-of-fit statistics [7]. If your primary goal is to make predictions, and you do not need to understand the role of each independent variable, you do not need to reduce or eliminate multicollinearity.

Overfitting a model is a condition where there are too many terms for the number of observations such that the statistical model begins to describe the random error in the data rather than the relationships between variables. In other words, the model is too complex and can produce misleading goodness-of-fit statistics, regression coefficients, and p-values [8]. As each sample has its own nuances, if the regression model becomes tailored to fit the random nuances of one sample it is unlikely to fit the random nuances of another sample reducing its generalizability outside the original dataset. Therefore, an overfit regression model describes the noise and is not applicable outside the sample.

## Discussion

The reviewed studies demonstrated a continued dependence on inferential statistical analyses with 408 studies (86.8%) reporting usage of regression or unadjusted statistical techniques. The most common test used was the independent samples t-test occurring in 45.7% of the reviewed studies. This is similar to other reviews of the medical literature. Although not specific to orthopaedic studies, Sato et al. in their review of 238 studies published in The New England Journal of Medicine found 224 studies (94%) utilized statistical methods with 31% of studies using a t-test [9]. Regression analyses were common in our review with 201 studies (42.8%) utilizing a regression analysis. This reflects the growing complexity of analyzed data in the orthopaedic literature and a desire for authors to attempt to control for potentially confounding variables that can be measured. The most common regression method was logistic regression utilized in 106 studies (22.6%).

None of the studies utilizing regression analyses reported checking all of the necessary underlying assumptions for the regression technique. This result supports our hypothesis. Of the most common regression, logistic regression, only 13 studies (12.3%) reported meeting even one assumption. Real et al. in their review of multivariable regression models from 500 studies randomly selected from MEDLINE also found that the most common regression method was logistic regression. Similarly, the authors found that the reporting of individual assumptions for all regressions was poor at only 26.2%. These authors also investigated the reporting of other aspects of statistical tests that are considered to be good statistical etiquette. The item that was reported most often was both crude and adjusted effects for models, but this was still only observed in 33.4% of studies analyzed. Reporting on the results of an interaction analysis was least frequent at only 18.5% [10].

The overall quality of statistics in the orthopaedic literature has been questioned in several studies, and unfortunately, our data contribute to this startling body of data. In their review of 100 representative orthopaedic studies, Parsons and associates found incorrect statistical tests, inefficient non-parametric tests, orphan p-values, and multiple comparisons without providing for any corrections [11]. These findings, in combination with loss to follow-up in clinical research, contribute to the difficulty of relying on significant findings in the orthopaedic literature [12].

The importance of assumptions lays in the fact that statistical methods were created starting with an assumption (e.g. there is a sample of independent observations following a normal distribution). Then, point estimators, intervals, and hypothesis tests are built from these assumptions. Therefore, the methods work well if the initial assumptions are true and that is why it is important to check them and report them. If the initial assumptions are not met, inferences can still be made, but the validity of those results may be doubtful. When testing hypotheses, running analyses on data that have violated the assumptions of the statistical test can result in both type 1 errors (false positives) and type 2 errors (false negatives), depending on the particular assumption violated. A violation can also result in overestimation or underestimation of the inferential measures and effect sizes. Such results are nonreproducible and in contradiction of the reproducible research movement [13,14].

Despite its importance, assumption checking is often not performed or reported, or both. It is likely that a number of factors play into this deficit. Many researchers are not aware or knowledgeable regarding what the assumptions are or how to check them. This lack of educated data analysts is compounded by the fact that most, if not all, statistical software packages do not automatically check assumptions; rather, they assume that these have been met. A computer program will produce an “answer” regardless of the appropriateness of that answer to a given type of data. Similarly, journal reviewers often are ill-equipped to raise questions regarding statistical assumptions and thus often assume that this important aspect has been adequately addressed. However, this study highlights that making an assumption about assumptions is unwise.

To complicate matters, there are often multiple ways to check any given assumption and many rely on subjective visual determinations of patterns (or lack thereof) for which researchers feel unqualified to interpret. Others argue that regression techniques are robust, working reliably even when their assumptions are not satisfied [14]. However, this does not excuse the researcher from checking assumptions for gross violations.

Many may look at an assumption violation as something the researcher has done wrong. On the contrary, the researcher is doing something very right and learning about their data structure, which helps to avoid potential statistical errors. This new information allows the researcher to select an alternative statistical method that better aligns with the data structure. In this situation, one should consider alternatives such as a nonparametric approach requiring fewer assumptions or a modern robust methodology [15,16].

Missing assumptions contribute to the already fragile data in orthopaedic studies across subspecialties. Khan and associates in their review of 48 randomized controlled trials in the sports medicine literature demonstrated a fragility index of 2, meaning that changing the outcomes of two patients from a non-event to an event in the treatment arm would change the statistical significance of a study [17]. Similar findings have been reported for other orthopaedic subspecialties with a fragility index of 2 reported in a review of the spine literature, a fragility index of 5 reported in a review of the trauma literature, and a low fragility index reported in randomized controlled trials in the hand literature [5, 18, 19].

Our data demonstrate the lack of reported assumptions in the published orthopaedic literature, which is consistent with the lack of understanding of medical statistics amongst orthopaedic residents [20]. It remains possible that the underlying assumptions were investigated, but not reported. However, as Hoekstra et al. demonstrated in their review of sample datasets provided for analysis to 30 researchers, assumptions were rarely checked for statistical tests. Follow-up interviews demonstrated a general ignorance regarding the need for assumptions when conducting inferential statistical testing [21].

The limitations of our analysis include the use of only a single orthopaedic journal with its own peer-review process. A more diverse selection of journals from the orthopaedic literature would strengthen our findings. Statistical assumptions were seldomly reported in one of the most prestigious of the orthopaedic surgery journals in which there are reviewers specific to methodology. However, it is the authors’ experience that statistical assumptions are frequently not reported throughout the orthopaedic literature. The proper analysis of data is of utmost importance in making appropriate conclusions and guiding patient care. Orthopaedic journals and their readers must be critical of presented statistics and aware of potential shortcomings. 

## Conclusions

Many recent studies published in JBJS depended on regression analyses to reach their conclusions, but none fully reported the necessary assumptions of these tests. Failure to report, and possibly conduct, statistical assumptions underlying regression analyses could call into question the findings of these statistical tests. We view our results as a call to greater education for orthopaedic researchers, as well as more rigorous and transparent reporting of the statistical methods employed in orthopaedic research with an emphasis on the importance of the peer-review process.
